# Microscopic changes in the multifidus muscle in people with low back pain associated with lumbar disc herniation

**DOI:** 10.1038/s41598-024-83373-9

**Published:** 2024-12-30

**Authors:** Shilpa Purushotham, Nathan Hodson, Carolyn Greig, Adrian Gardner, Deborah Falla

**Affiliations:** 1 Centre of Precision Rehabilitation for Spinal Pain (CPR Spine), School of Sport, Exercise and Rehabilitation Sciences, University of Birmingham, Birmingham, B15 2TT UK; 2 Department of Biomedical Sciences, School of Infection, Inflammation and Immunology, College of Medicine and Health, University of Birmingham, Edgbaston, Birmingham, B15 2TT UK; 3 Department of Sport and Exercise Sciences, Institute of Sport, Manchester Metropolitan University, Manchester, M15 6BH UK; 4 School of Sport, Exercise and Rehabilitation Sciences, University of Birmingham, Birmingham, B15 2TT UK; 5 MRC-Versus Arthritis Centre for Musculoskeletal Ageing and Health, University of Birmingham, Birmingham, B15 2TT UK; 6 National Institute for Health Research, Birmingham Biomedical Research Centre at University Hospitals Birmingham NHS Foundation Trust, Birmingham, B15 2TT UK; 7 The Royal Orthopaedic Hospital NHS Foundation Trust, Northfield, Birmingham, B31 2AP UK; 8 College of Health and Life Sciences, Aston University, Birmingham, B4 7ET UK

**Keywords:** Lumbar disc herniation, Low back pain, Multifidus, Fibre types, Pathological fibres, Clinical correlations, Muscle, Anatomy

## Abstract

Lumbar disc herniation (LDH) is a common degenerative condition causing low back pain (LBP) due to nerve compression. Previous studies show conflicting findings regarding the multifidus (MF) muscle’s microscopic changes in LDH patients. So, this study aimed to compare the affected MF to the adjacent MF on the ipsilateral and contralateral sides in LDH patients and examined correlations with clinical features of LBP. Four muscle biopsies were collected from each of 30 surgical participants. Immunohistochemistry was performed on tissue sections and imaged with an epifluorescence microscope. Data was analysed using a two-way ANOVA for muscle fibre cross-sectional area, perimeter, diameter, and composition, while pathological fibres were analysed using a one-way ANOVA. Pearson’s correlation was employed to examine MF microscopy associations with clinical features. Results revealed no significant differences between the affected MF and MF from other sites, though significantly more pathological fibres were present in the affected MF (p < 0.05). A weak but significant negative correlation was found between type I fibres and LBP clinical features, though no such correlations were observed for type IIA fibres. In conclusion, LDH primarily impacts the pathological status of the MF rather than fibre phenotype or size, and severity of clinical features is associated with the size of type I fibres.

## Introduction

Low back pain (LBP) is one of the most common musculoskeletal disorders, with nearly 80% of the population experiencing at least one episode in their lifetime^1,2^. The most common cause of LBP associated with radiculopathy is lumbar disc herniation (LDH) which is the most common degenerative disorder of the lumbar spine^3–6^. It is well-recognised that LDH can cause LBP and radicular leg pain secondary to nerve root compression^4,7,8^. The annual incidence of disc herniation is 5–20 cases/1000 adults and its prevalence is 31.9%^9,10^. The most common level of LDH is at L4-L5 (59%), followed by L5-S1 (30%) and then L3-L4 (9%) and the most clinically significant radiculopathies from nerve compression occur at these levels^11–13^.

Previous studies have examined the microscopic muscle morphology of the spinal extensor muscles in people with LBP, assessing both erector spinae and multifidus (MF) muscles^14–17^, although MF has been the most commonly investigated in LDH^18–23^ due to its unisegmental innervation^24^. Some of these studies have taken muscle samples at the same vertebral level of disc herniation, whereas some studies have sampled the affected muscle segment (innervated by the affected nerve root which is one vertebral level inferior to the affected disc level)^18,20,21^. The muscle is normally compared with a muscle sample from the asymptomatic side or compared with cadaveric samples as a control group^19,25^. However, conclusions vary widely on the structural changes in the muscle tissues concerning muscle fibre size, shape, area and composition,and also vary depending on the pain duration and severity^18–22^. Moreover, a recent systematic review reported heterogeneity in methodologies and insufficient evidence from MF microscopy on comparing the healthy and chronic LBP populations^26^.

To our knowledge, no study has compared microscopic muscle morphology at multiple adjacent vertebral levels in patients with single-level LDH. This is relevant because it would provide unique insight into whether structural compensation has occurred in the affected muscle’s adjacent and/or contralateral muscle segments, possibly due to functional adaptations due to pain such as muscle disuse, altered movement patterns, fear/pain avoidance, or deconditioning which may progressively worsen overtime and affect muscles at adjacent levels rather than just the affected muscle. Based on previous literature on the microscopic muscle morphology of MF in LDH^18,25^, we hypothesised that there would be atrophy of both type I (oxidative) and type II (glycolytic) fibres in the affected MF compared to unaffected MF on the ipsilateral and contralateral sides. Investigating the microscopic differences between the affected and the unaffected MF ipsilaterally at levels superior and inferior is important to be able to understand the extent and impact of any denervation injury. Therefore, in this study, we examined the microscopic muscle morphology of the lumbar MF at different vertebral levels on the affected side of disc herniation (ipsilateral) as well as on the contralateral side. Further, we examined the correlations of MF muscle microscopic morphology with clinical features including pain, perceived disability and the duration of pain in recognition that other factors and not just the presence of LDH may influence the extent of microscopic changes within the MF. The specific objectives were:

- To compare the muscle fibre cross-sectional area (CSA), fibre perimeter, narrow diameter (ND) and fibre type distribution of the affected MF with the adjacent muscle segments on the ipsilateral side and the contralateral side in people with LDH.
- To examine correlations between features of MF muscle microscopy and clinical features of LBP.

## Materials and methods

### Study design

This observational study was designed using ‘The Strengthening the Reporting of Observational Studies in Epidemiology’ (STROBE) guidelines^27^. The study was approved by the University of Birmingham research governance (RG22-049,ethics reference number ERN no. 22–0418) and ROH Research tissue bank (17/EM/0030). The study was conducted according to the Declaration of Helsinki and all methods were performed in accordance with relevant guidelines and regulations. Written informed consent was obtained from all study participants. Data was collected from April 2022- September 2023.

### Study setting

The research setting took place at a tertiary spinal centre in the UK for data collection and at the School of Sport, Exercise and Rehabilitation Sciences, University of Birmingham, UK for data extraction and analysis. Surgeons obtained the muscle samples and all the procedures on muscle samples were conducted by the primary researcher (SP).

### Participant eligibility criteria

#### Inclusion criteria:

- Age ≥ 18 years who are undergoing microdiscectomy surgery for LDH and consented to donate muscle tissue samples for research.
- A disc herniation at the lumbar level only with compression of the nerve root. Clinical clarification of the vertebral level in cases of lumbarised S1 and sacralised L5.
- Documented clinical or electrophysiological evidence of signs and symptoms of either nerve compression due to LDH in the sclerotome of the affected nerve root, muscle weakness in the myotome of the affected root or loss of sensation in the dermatome of the affected root.

#### Exclusion criteria:

- Disc herniation at any other vertebral level, to rule out the possibility of normal anatomical differences in muscle fibre composition at different vertebral levels.
- Structural deformities of the spine including scoliosis and spondylolisthesis.
- Revision cases/ patients undergoing repeat surgeries for the same issue.
- Medical comorbidities including
  o Diabetic neuropathy
  o Rheumatoid arthritis
  o A neurological diagnosis that could affect peripheral nerve function.

### Participants

Surgical participants were recruited for this study from the tertiary spinal centre, UK. Participants from the elective surgical list were screened for eligibility and the candidates with LDH scheduled for open microdiscectomy surgeries were consented by the research team of the tertiary spinal centre. The patient selection and recruitment were conducted by a team of research staff from the centre, including the Director of Research and Development, surgeons, and research nurses, and were blinded to the primary researcher. As per our knowledge, there was no bias or error in the patient selection or recruitment strategy. Given the study’s exploratory nature, a convenience sample of 30 people was recruited. This sample is comparable with a previous study examining microscopic changes within the MF in people with LDH^18,25^.

### Patient-reported outcome measures

Pain duration was documented and average and current pain intensity for LBP and lower limb pain were documented. Pain intensity was measured using a numerical pain rating scale (NPRS) (on a scale of 0–10; 0 = no pain and 10 = worst pain). Perceived disability was recorded using the Oswestry Disability Index (ODI) questionnaire^28^. These questionnaires have good validity, responsiveness and reliability for LBP and musculoskeletal pathology.^29–33^.

### Tissue sampling and storage

**Fig. 1:**
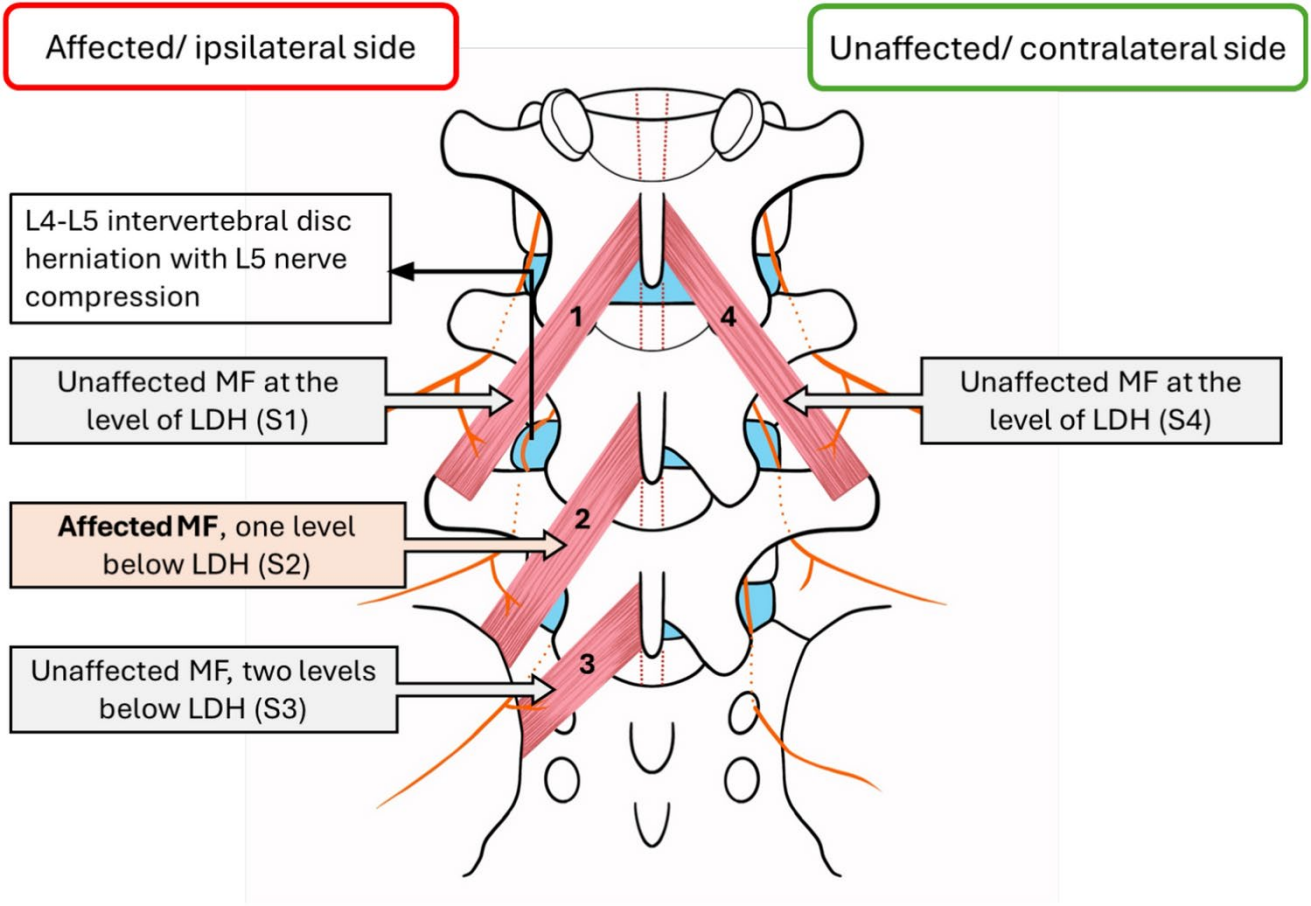
Schematic representation showing the different sites for obtaining multifidus muscle tissue samples. The figure shows the example of L4-L5 disc herniation and compression of the L5 spinal nerve. Multifidus muscle sampling Site 1 (S1), Site 2 (S2), Site 3 (S3) on the herniated side, Site 4 (S4) on the contralateral side.

The study methodology was based on well-established methods for examining muscle tissue^34–36^. Muscle biopsies were obtained by surgical experts who were further trained by an interventional radiologist for MF muscle biopsies both by clinical assessment and ultrasound-guided techniques. Four MF tissue samples were collected per patient, three from the affected side (MF sampling sites 1, 2, 3) and one from the contralateral side (MF sampling site 4) as shown in Fig. 1. All four samples were obtained intraoperatively. Site 1- at the vertebral level of disc herniation (unaffected muscle segment), site 2- one vertebral level inferior to the disc herniation (muscle segment affected by nerve compression), site 3- two vertebral level inferior to the disc herniation (unaffected muscle segment) and site 4- contralateral side at the vertebral level of disc herniation (unaffected muscle segment).

The muscle samples were embedded in an optimum-cutting temperature compound (Scigen Tissue Plus), frozen in Isopentane precooled by liquid nitrogen and stored at -80 degrees. The frozen samples were then transported in dry ice to the School of Sport, Exercise and Rehabilitation Sciences (UOB) under Material Transfer Arrangement (MTA no. 2136064) for sectioning and immunohistochemistry. The frozen tissue blocks were cut into 7 µm sections with a cryostat (Bright OTF model, manufactured by Bright Instrument Company Ltd., Huntington, England) at -25 degrees and a series of sections were collected. The sectioned specimens of each participant from all four sites were mounted on charged slide (Epredia™ SuperFrost Plus™ Adhesion slides, Fisher Scientific) and stored at -20 degrees before immunohistochemistry.

### Immunohistochemistry

The slides were air-dried for approximately 30–40 min at room temperature and rehydrated with two 5-min 1xPBS washes before being treated with antibodies. Immunofluorescent staining for MHC isoforms was carried out at room temperature by initially incubating the sections with 5% Goat serum diluted in 1xPBS for 30 min followed by three 5-min washes in 1xPBS. The sections were then incubated with primary antibodies specific to MHC I (BA.D5 IgG2b), MHC IIA (SC.71 IgG1) and MHC IIX (6H1 IgM) (Developmental Studies Hybridoma Bank, Iowa City, IA, USA) without dilution for 90 min followed by three 5- minute washes in 1xPBS. Next, they were incubated with secondary antibodies Gt anti-Ms IgG2b, Alexa Fluor 350 (1:250) (product code A21140), Gt anti-Ms IgG1, Alexa Fluor 488 (1:250) (product code A21121) and Gt anti-Ms IgM, Alexa Fluor 555 (1:250) (product code A21426) diluted in 1xPBS for 60 min in dark followed by three 5-min washes in 1xPBS. This was followed by incubation with Wheat Germ Agglutinin, Alexa Fluor™ 350 Conjugate (thermofisher.com) (1:100) diluted in 1xPBS for fibre laminin for 20 min in dark. The slides were washed again with 1xPBS twice, mounted using ProLong™ Gold Antifade reagent, allowed to dry in the dark at room temperature overnight and then stored at -20 degrees.

### Microscopy and data extraction

Stained sections were observed under fluorescent microscopy (EVOS M5000 Imaging system) and images were captured at × 20 magnification. All samples for each participant were imaged on same slide with identical image settings. The images were analysed using Fiji ImageJ software for all the outcome measures including fibre CSA, fibre diameter/narrow diameter (ND), fibre circumference or perimeter and fibre type composition. Fibres with a circularity of less than 0.65 were removed prior to statistical analysis^37^. To analyse the microscopic morphological characteristics of the entire MF muscle from the representative sample, an average of 197 ± 9 fibres/sample was counted per sample for the composition (from sites S1, S2, S3 and S4) and an average of 120 ± 27 fibres/sample was measured for the CSA (µm^2^), ND (µm) and perimeter (µm). Relative Cross sectional Area (RCSA) was calculated based on CSA and the number of muscle fibres for each fibre type at different sites using the formula:

RCSA type I = mean CSA type I x no. of type I/ sum (mean CSA type i x no. of type i); i = different fibre types.

The recommended sample size required for measurements is a minimum of 50 fibres for type I and II fibres per individual^38^. Fibres expressing only MHC I (BA.D5) were categorised as type I, fibres expressing only MHC IIA (SC.71) as type IIA and fibres expressing strong intensities of MHC IIX (6H1) were categorised as type IIX. Fibres expressing intermediate intensities of both MHC I (BA.D5) and MHC IIA (SC.71) were classified as type I/IIA hybrid fibres. Fibres expressing intermediate intensities of MHC IIA (SC.71) and strong intensities of MHC IIX (6H1) were classified as type IIAX hybrid fibres.

### Data analysis

Data extracted for all the outcome measures and demographic data, including pain and disability scores, were tabulated in Microsoft Excel. Shapiro–Wilk and the Levene tests were used to confirm data normality and ensure homogeneity of variance, respectively. Given that these conditions were satisfied, parametric tests were used. Statistica (StatSoft, Germany) was used to analyse the differences between microscopic features of MF at the different sites for all four outcome measures. A two-way ANOVA was performed with the site of muscle samples and fibre types as within-subject factors (4 & 3 levels, respectively). Significance was set at p < 0.05 and a confidence interval of 95%. When significance was identified, a post hoc Newman-Keuls test was used to determine the direction of the significant differences. To analyse the pathological fibres, a one-way ANOVA was performed with the site of the muscle samples as within-subject factor (4 levels). If the assumption of sphericity (Mauchly’s test p < 0.05) was violated, a Greenhouse–Geisser correction was used.

The correlations between the outcome measures of muscle microscopy and clinical parameters as well as participant age were measured in SPSS using bivariant, one-tailed Pearson’s correlation coefficient. The correlations were measured between -1 to 1. ‘- ‘represents negative, ‘ + ’ represents positive and ‘0’ represents no correlations, with the strength of the relationship indicated as very weak (0.01–0.19), weak (0.2–0.39), moderate (0.4–0.59), strong (0.6–0.70), very strong (0.8–0.99) and perfect (1.0).

## Results

Data from 30 participants (11 men, 19 women) were included in the study. Participants had a mean age of 46.9 ± 15 years (range, 21–85), and their mean BMI was 31.7 ± 5.7 kg/m^2^ (range, 20.5- 41.9). Details on the vertebral level of LDH, pain-related factors and disability are provided in Table 1. LDH was present at vertebral levels L3-L4, L4-L5 and L5-S1 in 3, 12 and 15 participants respectively. 15 participants had LDH on the right side whereas 12 participants had it on the left side and 3 participants had bilateral disc herniation but were symptomatic only on the left side (affected side). All participants experienced LBP (except one) and radiculopathy lower limb pain secondary to neuronal compression from disc herniation. The mean duration of LBP was 45.5 ± 56.6 months (range, 0–240), and the mean of their average and current LBP intensity was 5.4 ± 2.9 and 5.0 ± 2.9, respectively, on a scale of 0–10. The mean Oswestry Disability Index (ODI) was 52.6 ± 17.2% (range, 20–96).

**Table 1:** Summary of clinical features and patient-reported outcome measures (n = 30).

Clinical features and patient-reported outcome measures		Values
LDH level	L3-L4	3
	L4-L5	12
	L5-S1	15
LDH side	Right	15
	Left	15 (3 b/l)
LBP	Rate (%)	96.66
	Average intensity (mean ± SD)	5.4 ± 2.9
	Current intensity (mean ± SD)	5.0 ± 2.9
	Pain duration (months)	45.5 ± 56.6
LL pain	Rate (%)	100
	Average intensity (mean ± SD)	6.8 ± 2.8
	Current intensity (mean ± SD)	6.6 ± 2.7
	Pain duration (months)	22.3 ± 19.3
ODI scores (%)		52.6 ± 17.2

LDH, lumbar disc herniation; LBP, low back pain; LL, lower limb; ODI, Oswestry Disability Index; L, lumbar; S, sacral

**Fig. 2:**
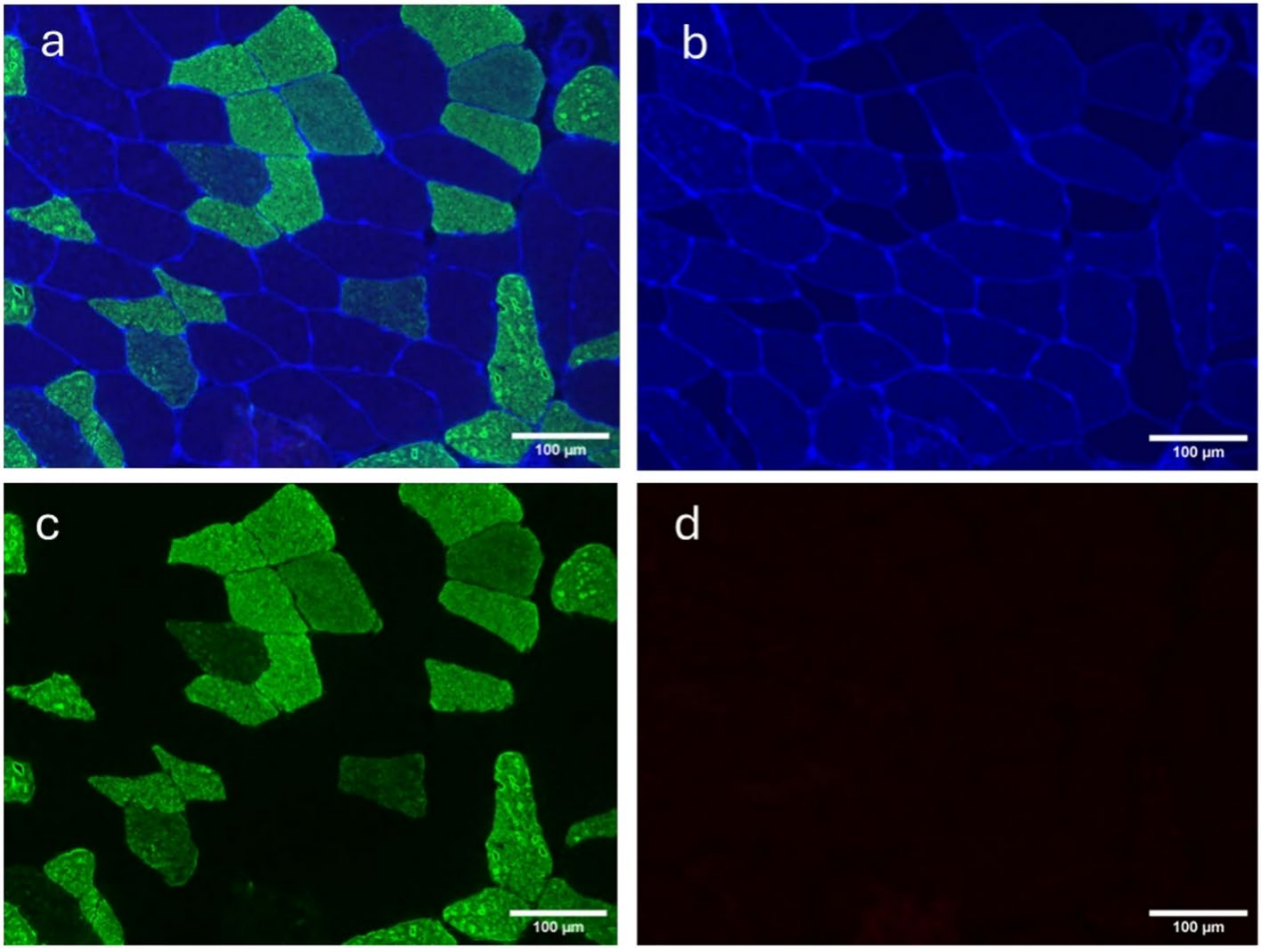
Representative immunofluorescent microscopic image of a lumbar MF sample. MF cross-sections incubated with primary antibodies against MHC I (blue), MHC IIA (green), MHC IIX (red) and laminin (blue). Image a, fibres are seen in all channels (blue, green, and red); Image b, type I fibres seen in the blue channel; Image c, type IIA fibres seen in the green channel; Image d, type IIX fibres seen in the red channel. Note: This microscopic section presented as image d has no fibres in the red channel. Type IIX fibres were observed only in a few participants and very small numbers. MF, multifidus; MHC, myosin heavy chain. Scale bar 100 µm.

A representative sample of MF muscle from one of the participants is shown in Fig. 2.

Statistical analysis for outcome measures was done for type I and type IIA fibres only, as type IIX fibres, type I/IIA hybrid fibres and type IIAX hybrid fibres were not present in all the muscle samples (and participants) and hence there was substantial missing data for these fibre types which would not be appropriate to impute. Further, muscle samples from site 3 (S3) were missing from 5 participants (no S3 sample collection from 3 participants due to surgical difficulty in obtaining a biopsy from this site and insufficient tissue samples in 2 participants). This minimal loss of data (~ 4% of the total dataset) was dealt by the Statistica software without compromising the participant number.

### Fibre cross-sectional area

**Fig. 3:**
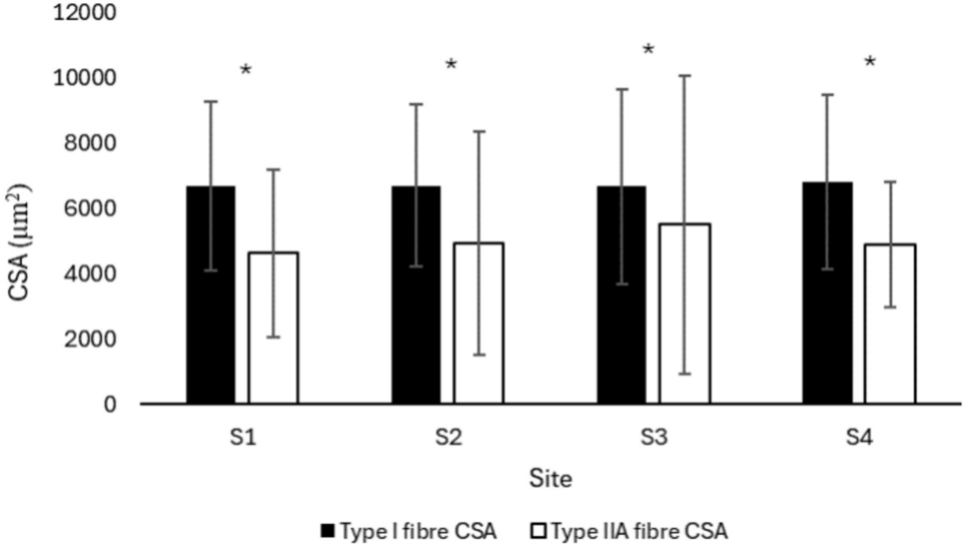
CSA of type I and type IIA fibres of lumbar MF muscle at different sites. Values are represented as mean ± SE. CSA, cross-sectional area; MF, multifidus; S, site; SE, standard error. *p < 0.001, type I vs. type IIA fibres.

There was no statistically significant difference in the fibre CSA of type I and type IIA fibres of the MF when compared at different sites of the lumbar region (F = 0.1986, p = 0.89). However, there was a statistically significant difference between the CSA of type I and type IIA fibres (F = 19.2871, p < 0.001). Post hoc analysis revealed a greater CSA for type I fibres compared to type IIA fibres in MF (p < 0.001) regardless of the lumbar vertebral level/site (F = 0.2320, p = 0.87) (Fig. 3).

### Fibre circumference/perimeter

**Fig. 4:**
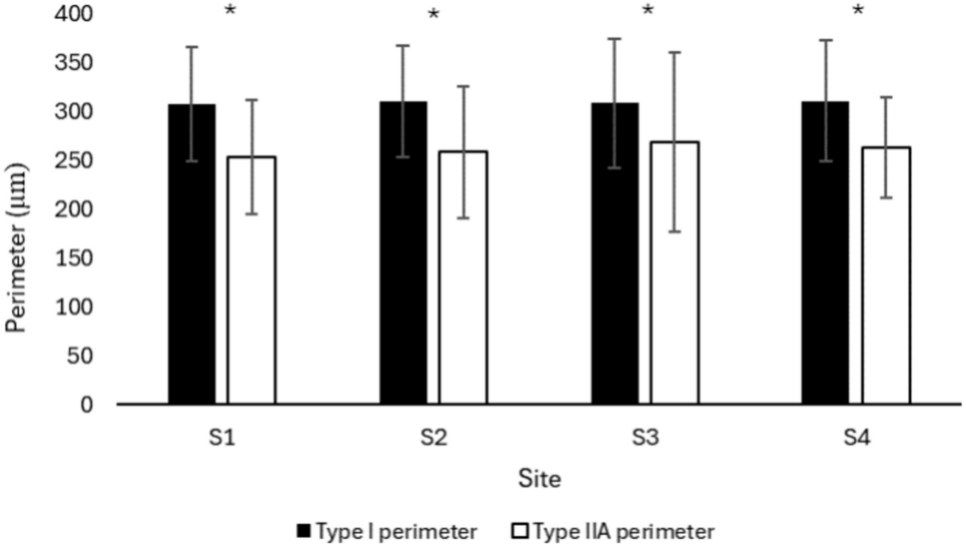
Perimeter of type I and type IIA fibres of lumbar MF muscle at different sites. Values are represented as mean ± SE. MF, multifidus; S, site; SE, standard error. *p < 0.001, Type I vs. type IIA fibres.

There was no statistically significant difference in the perimeter of type I and type IIA fibres of the MF when compared at different sites of the lumbar region (F = 0.183, p = 0.90). However, there was a statistically significant difference between the perimeter of type I and type IIA fibres (F = 31.929, p < 0.001). Post hoc analysis shows a greater perimeter for type I fibres compared to type IIA fibres in MF muscle (p < 0.001) regardless of the lumbar vertebral level/site (F = 0.133, p = 0.94) (Fig. 4).

### Fibre diameter/narrow diameter

**Fig. 5:**
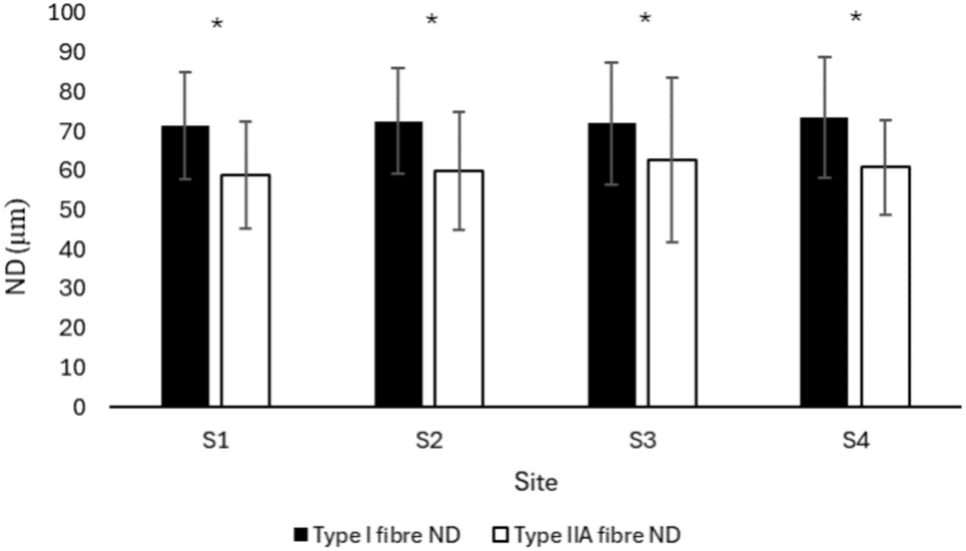
ND of type I and type IIA fibres of lumbar MF muscle at different sites. Values are represented as mean ± SE. ND, narrow diameter; MF, multifidus; S, site; SE, standard error. *p < 0.001, Type I vs. type IIA fibres.

There was no statistically significant difference in the ND of type I and type IIA fibres of the MF when compared at different sites of the lumbar region (F = 0.267, p = 0.84). However, there was a statistically significant difference between the ND of type I and type IIA fibres (F = 35.686, p < 0.001). Post hoc analysis shows a greater ND for type I fibres compared to type IIA fibres in MF muscle (p < 0.001) regardless of the lumbar vertebral level/site (F = 0.173, p = 0.91) (Fig. 5).

### Percentage fibre type composition (%)

**Fig. 6:**
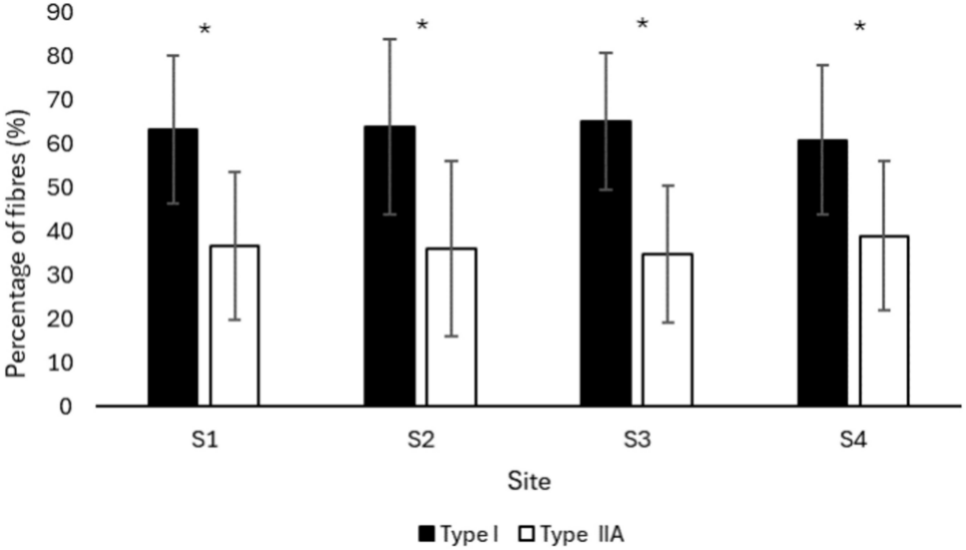
Percentage of type I and type IIA fibres of lumbar MF muscle at different sites. Values are represented as mean ± SE. MF, multifidus; S, site; SE, standard error. *p < 0.001, Type I vs. type IIA fibres.

There was no statistically significant difference in the percentage fibre composition of the type I and type IIA fibres of the MF muscle when compared at different sites of the lumbar region (F < 0.0005, p = 1.0). However, there was a statistically significant difference between the percentage of type I fibres compared to type IIA fibres (F = 132.71, p < 0.001). Post hoc analysis shows a greater percentage of type I fibres compared to type IIA fibres in MF (p < 0.001) regardless of the lumbar vertebral level/site (F = 0.591, p = 0.62) (Fig. 6).

Further, the percentage composition of fibre types was analysed for all fibre types at different sites of lumbar MF. Since type IIX, and hybrid fibres I/IIA and IIAX were present only in small numbers in some muscle samples, no statistical analysis was performed on this data. However, percentage composition was calculated to understand the fibre type distribution, as shown in Table 2. It was noted that type I/IIA fibres were present in all the participants but not all sites assessed.

**Table 2:** Showing fibre type composition of MF muscle at different sites in the LDH.

Fibre comp (%)	S1	S2	S3	S4
Type I	61.24 ± 16.65	61.92 ± 20.89	63.14 ± 15.62	59.42 ± 16.99
Type I/IIA	1.61 ± 3.02	2.30 ± 3.09	2.96 ± 2.87	2.45 ± 2.37
Type IIA	35.24 ± 16.28	34.32 ± 18.70	33.51 ± 15.34	37.80 ± 16.15
Type IIAX	1.39 ± 1.96	1.12 ± 2.48	0.14 ± 0.40	0.24 ± 1.02
Type IIX	0.49 ± 1.37	0.32 ± 1.62	0.22 ± 0.82	0.07 ± 0.31

Values presented as Mean ± SD.

Table 2 shows % fibre distribution for all fibre types and indicates a small tendency for a higher % of type IIAX and type IIX fibres at the affected MF (site 2) and the unaffected MF above on the same side (site 1) compared to the unaffected MF below on the ipsilateral side (site 3) and contralateral side (site 4).

### Relative area covered by each fibre type (relative cross-sectional area RCSA)

The results indicate a small tendency for a higher percentage of RCSA for both type IIAX and type IIX at site 2 (affected MF) compared to the other sites (Table 3).

**Table 3:** Percentage RCSA of fibre types in MF at different sites in the LDH.

RCSA (%)	S1	S2	S3	S4
Type I	69.16 ± 17.96	68.728 ± 20.48	69.31 ± 18.55	65.55 ± 19.39
Type I/IIA	1.32 ± 2.50	1.8 ± 2.61	2.3 ± 2.56	2.08 ± 3.07
Type IIA	28.34 ± 17.22	27.79 ± 18.75	28.0 ± 17.52	32.19 ± 17.92
Type IIAX	0.54 ± 1.32	0.84 ± 2.41	0.14 ± 0.44	0.15 ± 0.60
Type IIX	0.6 ± 1.94	0.83 ± 4.30	0.23 ± 1.12	0.009 ± 0.05

Values presented as Mean ± SD.

### Pathological fibres

**Fig. 7:**
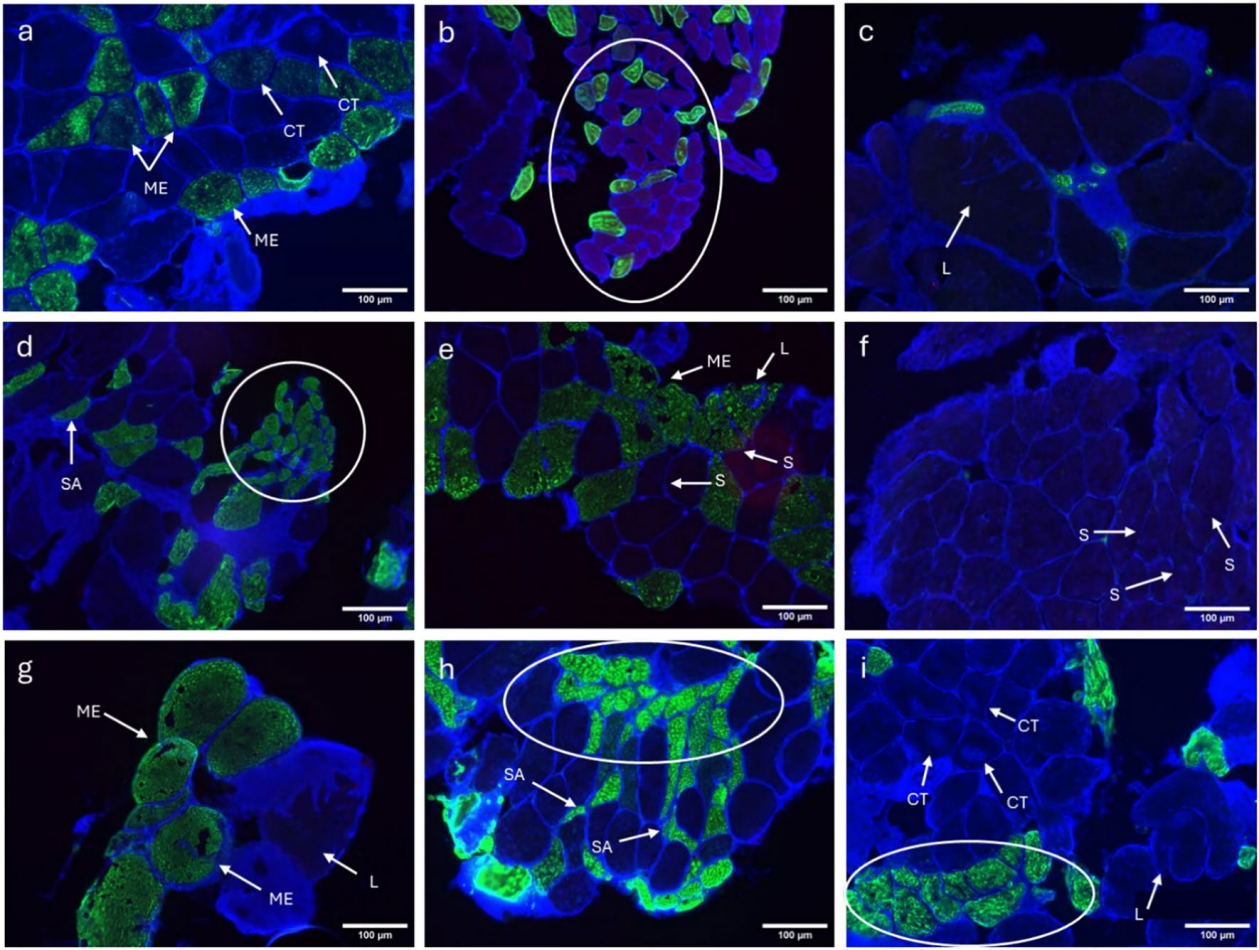
Representative immunofluorescent microscopic image of a lumbar MF muscle sample showing pathological fibres. (**a**) arrows pointing at core-targetoid and moth-eaten fibres; (**b**) showing fibre atrophy; (**c**) showing lobulated fibres; (**d**) fibre grouping and atrophy and arrow indicating the small angulated fibres; (**e**) arrows pointing at moth-eaten, lobulated and split fibres; (**f**) arrows showing split fibres; (**g**) arrows pointing at moth-eaten and lobulated fibres; (**h**) fibre grouping atrophy and small angulated fibres; (**i**) showing fibre grouping of moth-eaten fibres and arrows pointing at core-targetoid fibres. CT, core-targetoid; ME, moth-eaten; L, lobulated; SA, small angular; S, split fibres; Scale bar 100 µm.

Abnormalities in the fibres were observed in most of the muscle samples, and though present inconsistently at different sites, they were observed relatively more in the affected muscle (site 2). Pathologies like moth-eaten appearance and small-angulated fibres were frequently seen in MF samples. Core targetoid fibres, lobulated fibres, split fibres, group atrophy of fibres and fibre-type groupings (due to denervation and reinnervation) were also observed in some muscle samples. These pathologies are shown in the representative samples of the MF affected by nerve compression/ LDH in Fig. 7.

**Fig. 8:**
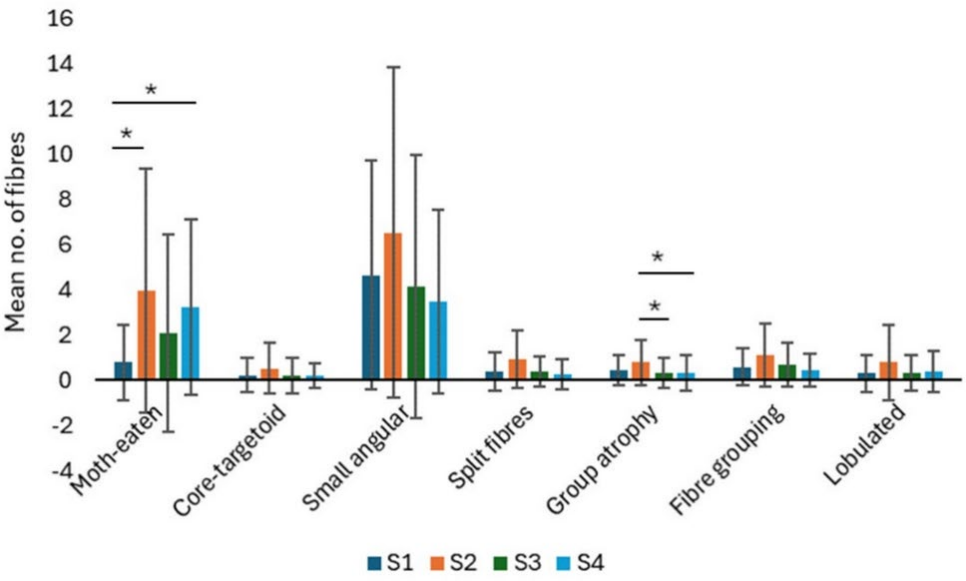
Mean number of pathological fibres seen at different sites (S) in lumbar MF muscle affected by LDH. type I and type IIA fibres of lumbar MF muscle at different sites. Values are represented as mean ± SE. MF, multifidus; S1- unaffected MF (upper segment on the ipsilateral side), S2- affected MF, S3- unaffected MF (lower segment on the ipsilateral side), S4- unaffected MF contralateral side; SE, standard error. *p < 0.05.

Pathological fibres were noted at S1 in 27 participants, at S2 in all 30 participants, at S3 in 25 and at S4 in 28 participants. Overall, there is a tendency for an increase in pathological fibres at S2 as seen in Fig. 8.

The results show a statistically significant difference in the mean number of moth-eaten fibres (F = 4.862, p = 0.004) when compared at different sites. The post hoc analysis showed that this difference was significant between the unaffected MF (S1) and affected MF (S2) ipsilaterally (p = 0.025) and between the unaffected MF (S1) and contralateral unaffected MF (S4) (p = 0.026). There was a significant difference in the mean number of group atrophy of fibres (F = 4.097, p = 0.009) when compared at different sites. Post hoc analysis showed this difference was significant between affected MF (S2) and unaffected MF ipsilaterally (S3) (p = 0.035) and between the affected MF (S2) and contralateral unaffected MF (S4) (p = 0.049) for group atrophy. For fibre type grouping (F = 2.913, p = 0.039), split fibres (F = 3.574, p = 0.029) and small angular fibres (F = 3.769, p = 0.025) there was a significant difference between the sites, but this significance was not maintained after the post hoc analysis and correction for multiple comparisons. There was no significant difference in the mean number of core-targetoid fibres (F = 1.062, p = 0.36) and lobulated fibres (F = 1.574, p = 0.219) among the different sites.

### Correlation with clinical features

Pearson’s correlation coefficient was used to examine the correlation between type I and type IIA fibre parameters (fibre CSA, perimeter, ND, and composition) with clinical features of pain (average LBP intensity, current LBP intensity i.e. at the time of biopsy and duration of LBP) and disability (ODI score). As there were no statistically significant differences in outcome measures across different MF sites for both type I and IIA fibres, an average was calculated across all 4 sites for type I and IIA fibre parameters for each participant.

**Fig. 9:**
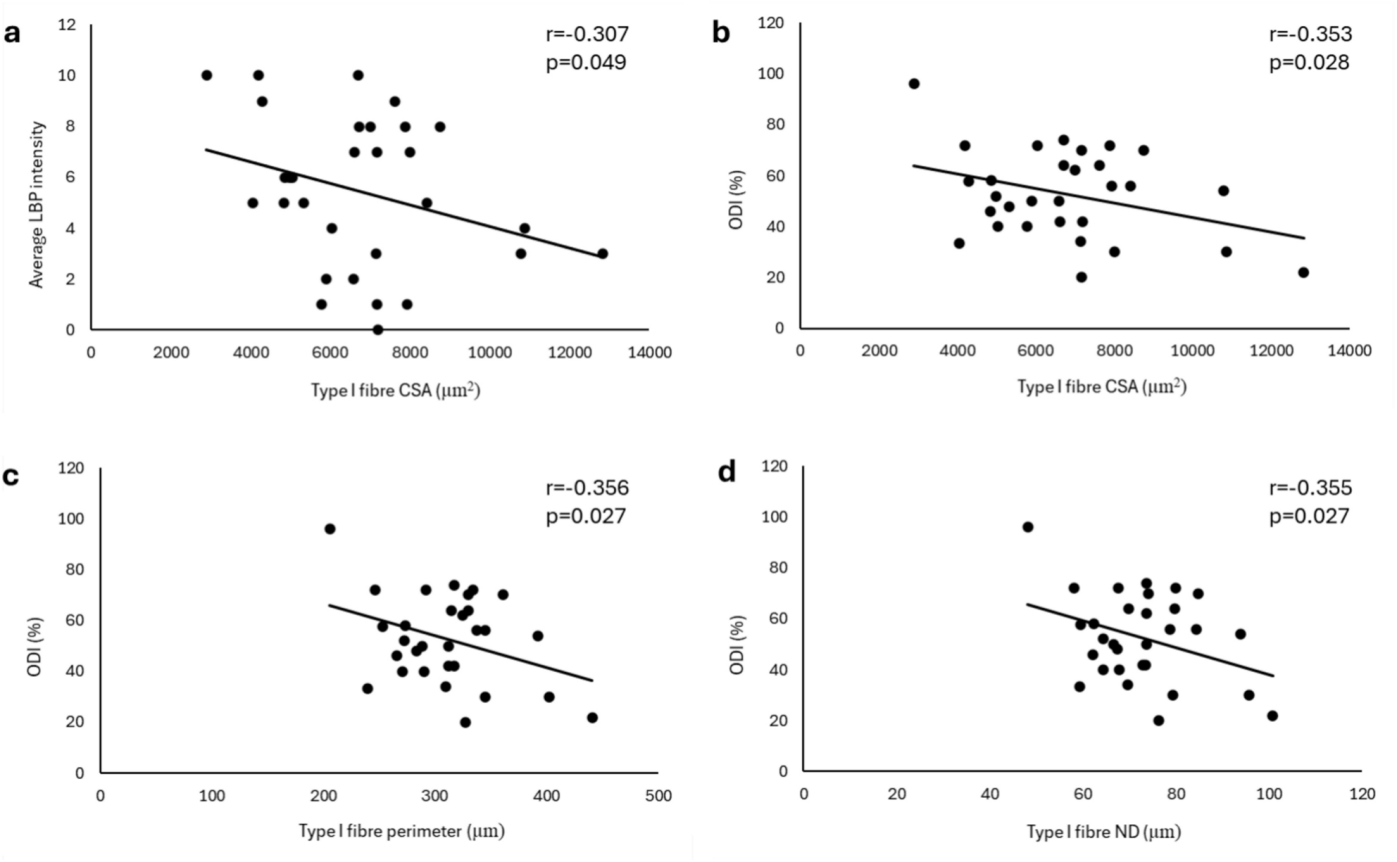
Pearson correlations showing weak but significant negative correlation between (**a**) Type I fibre CSA and average intensity of LBP. (**b**) Type I fibre CSA and ODI score. (**c**) Type I fibre perimeter and ODI score. (**d**) Type I fibre ND and ODI score. LBP, low back pain; CSA, cross-section area; ND, narrow diameter; ODI, Oswestry Disability Index.

Weak but significant negative correlations were found between the CSA of type I fibres and average LBP intensity (r = -0.307, p = 0.049) (Fig. 9a) and ODI score (r = -0.353, p = 0.028) (Fig. 9b). Weak but significant negative correlations were also found for type I fibre perimeter and ND with ODI (r = -0.356, p = 0.027) (Fig. 9c) and (r = -0.355, p = 0.027) (Fig. 9d) respectively. None of the clinical features of LBP were associated with age (all p > 0.05).

No correlation were noted for type I fibre parameters with pain duration. No correlations were noted for the type IIA fibre parameters with any clinical feature of LBP (pain intensity, duration and ODI).

## Discussion

This study examined the differences in MF muscle morphology microscopically at different vertebral levels in patients with LDH. The main findings of our investigation contradict our hypothesis and show that there were no significant differences in the MF microscopic morphology when the affected muscle segment was compared with the adjacent muscle segments on the same side of disc herniation and the contralateral side for the main outcome measures (fibre CSA, perimeter, ND and composition). However, there were significant differences between the type I and type IIA fibres in all these outcome measures with type I fibres having greater CSA, perimeter, ND and distribution compared to type IIA fibres regardless of MF location.

In addition, we observed the appearance of pathological fibres such as moth-eaten appearance, core-targetoid fibres, split fibres, lobulated fibres and atrophy of fibres and fibre grouping in most of the muscle samples. These pathological changes, although seen at all sites, were significantly more notable in the affected muscle (S2) and are indicative of negative changes in muscle architecture in those with LDH.

As a further finding, some fibre characteristics were associated with the clinical presentations of the participants with LDH. There was a negative correlation between the type I fibre CSA and average LBP intensity and, a negative correlation for type I fibre parameters (CSA, perimeter and ND) with perceived disability. These results indicate that a higher pain intensity and disability is related to a decrease in the type I fibre area and that higher disability is associated with lower type I fibre perimeter and diameter in the lumbar MF muscle. There were no correlations noted for type IIA fibres with the pain and disability related factors.

To our knowledge, there are no studies examining the MF muscle at multiple locations on the affected side in patients with disc herniation. In contrast to our hypothesis, we observed no significant difference in the muscle microscopy of the affected MF with the unaffected MF which is consistent with some of the previous studies^16–18,39,40^. Yoshihara et al.^18^ compared the MF muscle on the affected and unaffected side at two levels in people with LDH and showed no significant level-to-level difference in fibre size. However, they noted atrophy of fibres in the affected muscle when compared with the contralateral side at the same level. Research by Agten et al.^16^, showed no significant difference in the MF muscle CSA, percentage composition and RCSA between people with chronic LBP and an asymptomatic population. Similarly, Ford et al.^39^, did not find any morphological differences in MF. Likewise, Crossman et al.^17^, showed no significant differences in the paraspinal muscle microscopic parameters when comparing people with chronic LBP to asymptomatic individuals, however, this study assessed the erector spinae muscle and only in males. Another study showed no significant difference in the fibre size between people with chronic LBP and asymptomatic individuals, but in this study, MF in those with chronic LBP was compared with the erector spinae from the control group^41^.

Despite research showing similar findings to our study, some studies have reported smaller type I and type II fibre size, CSA and diameter in the MF muscle when the affected segment or the segment at the level of LDH was compared with the unaffected side or to that of a control group^14,18,21,42^. Some studies have contradictory findings showing a lesser percentage of type I fibres^14,41^ or a greater percentage of type I fibres^18,42^ on the affected side when compared with unaffected side or a control group. Likewise, Agten et al.^16^, reported an increase in the number of type I fibres and a trend towards higher and lower CSA for type I and IIX fibres respectively in people with chronic LBP compared to healthy controls but these observations were made in the erector spinae muscle. On the contrary, some studies reported larger fibre size and diameter in MF at the herniation level^19,43^ but these studies used cadaveric muscles to compare which may not be histologically similar to the living population, as atrophy of muscles is expected in cadaveric samples. On the other hand, there have been inconsistencies concerning type II fibres in MF with some studies showing a lesser % of type II fibres in LDH^19^ or a lesser % IIA and a greater % of type IIB in chronic LBP^15^. Overall, the findings of our research are comparable to a recent systematic review reporting no significant difference between the microscopy of paraspinal muscles, particularly erector spinae, between a LBP and pain-free population^26^.

On comparing the fibre characteristics between the type I and type II fibres, the results of this study show that the outcome measure parameters of type I fibres are significantly greater than type IIA fibres regardless of the location of the lumbar MF muscle. Similar observations were made on MF in several other studies, where they reported significantly greater type I fibre size^18,44,45^, diameter^19^^,^ CSA^45^ and number^19,39,46^ when compared to type II fibres. Similar observations have been made for the erector spinae^16,17,47^. On the contrary, two studies showed a significantly higher percentage of type II^14^ or IIB^41^ fibres in MF compared to type I in a cohort of people with LBP. However, much of the evidence as discussed suggests higher type I fibre parameters compared to type II fibres in back muscles which can be attributed to their well-known role in postural stability^35,48,49^.

On comparing all fibre types, the findings in this study show that the percentage fibre composition of type IIX fibres along with hybrid fibres, type I/IIA and IIAX were small compared to type I and type IIA fibres. A similar finding of more type IIA fibres compared to type IIX fibres in MF was presented in a previous study^43^. It was also observed that the type I/IIA fibres were consistently seen in all the participants (though not consistent with the location of the muscle) but not the type IIX or type IIAX fibres. The study results show a tendency for relatively higher percent fibre composition and RCSA of type IIX and type IIAX fibres in the affected muscle compared to MF from other sites. This is in line with the findings from Mannion et al.^41^, and these findings may be indicative of fibre-type changes or conversions. The presence of type I/IIA hybrid fibres in all the participants may suggest a possible glycolytic shift with gradual fibre type conversions from type I to type IIA fibres and from type IIA to IIX due to pain, muscle disuse and nerve injury in the affected muscle segment^50,51^.

There were observations of abnormal or pathological fibres in all the sites of MF but more commonly in the affected site. While the results show a higher tendency for all the pathological variations in the affected MF, these abnormalities were statistically significant for moth-eaten fibres, group atrophy of fibres, split fibres, small angular fibres, and fibre grouping. Similar pathological changes were observed in previous LDH and LBP studies and they are likely indicative of nerve injury and excessive muscle strain^18,20–22,40,44,47^. Rantanen et al*.*^20^*,* followed up with the LDH participants 5 years postoperatively and reported that the frequency of pathological changes was found to decrease in patients with good recovery signs. They further proposed that axonal injury along with inactivity contributed to pathological changes, along with selective atrophy of type II fibres. Nonetheless, it is also reassuring that these changes are potentially reversible or can be reduced with adequate treatment measures like surgery and physiotherapy^20,40^.

The size of type I fibres showed negative correlations with pain intensity and disability. This indicates that with an increase in the average pain intensity and disability, the type I fibre size decreases. No correlations were observed between fibre size and pain duration, and this is comparable to the observation made by Mannion et al.^47^. However, unlike the results of this study showing no correlation between the percent fibre composition and pain duration, Mannion et al.^47^, observed a negative correlation between these variables, i.e., the longer the pain duration, a lesser number of type I fibres and a greater number of type IIX fibres. This study did not find any correlations between microscopic features of the MF and pain duration, likely because of the range of pain duration for the participants included in this study. As the participants were from the elective surgical list who experienced long-term pain, the results show definitive structural changes (like pathological fibres). Correlations with pain duration might have been evident if, along with chronic pain, the study included people with acute pain (inflammatory-related changes) where microscopic structural changes may not yet be apparent. The current study showed no correlations between the type IIA fibre parameters and clinical features, which may further support the argument that there is no atrophy or decrease in type IIA fibres in the muscle with a possible tendency towards glycolytic shift due to altered patterns of muscle activation due to pain.

### Methodological considerations

Muscle samples were collected by surgeons, and they were trained for precise tissue collection by an interventional radiologist. However, there was no specific direction to obtain superficial or deep MF fibres. Further, the muscle biopsies were collected at three lumbar vertebral levels of LDH, and the inclusion criteria were not restricted to a single vertebral level for consistency. However, this limitation is addressed within the study as irrespective of the LDH level, samples were collected from adjacent vertebral levels for comparison (within subject) in addition to the contralateral side.

The differences in the muscle microscopy between males and females were not analysed due to a mismatch in the participant numbers and small sample size. However, since the muscle comparison was within the subject, the results are valid regardless of sex or other factors such age or BMI. In addition, this study did not document details on the physical activity level of the population investigated. This may have revealed how the level of physical activity impacts the fibre changes. Microscopically, muscle strain, which can be detected as muscle fibre atrophy and split fibres, was quantified and reported in the study. However, other indicators of muscle strain, like infiltration of inflammatory cells and connective tissue fibrosis, were not identified or investigated as they depended on the cause and duration of muscle strain/injury and were beyond the scope of this study. Lastly, although denervation may have affected adjacent segments in the form of pathological changes as seen in this study, it can be argued that the unaffected muscle segments act as more robust controls as this eliminates the interindividual variations, including anthropometric and demographic differences along with differences in physical activity levels and certainly a more valid control comparison than a cadaver.

## Conclusion

In contrast to what we expected, there were no differences in muscle fibre CSA, perimeter, ND and % fibre composition of the lumbar MF between that assessed at the level of LDH and neighbouring sites as well as the contralateral side. However, there were more pathological fibres in the affected MF, suggesting LDH may mainly impact skeletal muscle fibre pathology status rather than phenotype and/or size. Higher pain intensity and disability were associated with smaller type I fibres indicating that the severity of clinical features affects the size of oxidative fibres.

## Data Availability

The article contains all the data underlying this study’s results; no additional data source is required. Raw data values that support this study’s findings are available on request from the corresponding author.
